# Electricity distribution networks resilience in area exposed to salt dust: Fragility curve modeling of insulators, Monte Carlo-based resilience assessment, and enhancement measures

**DOI:** 10.1016/j.heliyon.2024.e35804

**Published:** 2024-08-06

**Authors:** Amin Dadashzade, Hossein Bagherzadeh, Masood Mottaghizadeh, Tohid Ghanizadeh Bolandi, Mohammad Hassan Amirioun, Maryam Majidzadeh, Sajjad Golshannavaz, Farrokh Aminifar

**Affiliations:** a School of Electrical and Computer Engineering, College of Engineering, University of Tehran, Tehran, Iran; b Faculty of Electrical and Computer Engineering, Urmia University, Urmia, PO Box 5756151818, Iran; c Department of Electrical Engineering, Shahreza Campus, University of Isfahan, Iran; d Department of Electrical Engineering, Technical and Vocational University (TVU), Tehran, Iran

**Keywords:** Fragility curve, Failure probability, High impact low probability (HILP) events, Insulator, Monte Carlo-based resiliency assessment, Salt dust

## Abstract

Resilience of the power system against natural disasters is a vital need for sustainable energy supply. As a result of global warming, lakes and rivers have dried out, resulting in dust hubs that threaten the normal operation of outdoor power system equipment. Unlike other events like hurricanes and blizzards, the impact of extreme salt dust on power system insulator failures and network resilience in affected areas remains unexamined. In this paper, to avoid power curtailment caused by insulators breakdown in electricity distribution networks, the resiliency assessment and enhancement of these networks against salt dust is investigated. Failure mechanism analysis of insulators and fragility curves extraction of them in face of salt pollution and relative humidity are done using mathematical modelling and experimental tests to extract the breakdown probability; Experimental tests are conducted in the High Voltage Laboratory, University of Tehran (HVLUT) and a novel method is proposed to extract 3-dimensional fragility curves of insulators. A Monte Carlo-based resiliency assessment method is then employed to obtain resiliency curve against the salt dust. Some suitable indicators are introduced for this purpose. In addition, several resiliency enhancement measures are proposed and ranked using a benefit to cost ratio (BCR) index. Numerical simulations are conducted on two real distribution feeders in a distribution grid around Urmia Salt Lake, Iran. Numerical results confirm the effectiveness and applicability of the proposed method.

## Highlights

• A probabilistic method for resiliency assessment and enhancement of distribution networks against extreme salt dust.
• Modeling the fragility curves of insulators against salt pollution and relative humidity.
• Deriving 3-D fragility curves of insulators.
• Identifying and prioritizing the potential resiliency enhancement countermeasures using benefit to cost ratio (BCR) method.

**Table:** Nomenclature

Acronyms	
*HILP*	High impact low probability
*REI*	Restoration efficiency index
*RI*	Resiliency index
*BCR*	Benefit to cost ratio
*VI*	Vulnerability index
*DI*	Degradation index
*RH*	Relative humidity percentage
*3-D*, *2-D*	Three-dimensional, two-dimensional
Parameters	
*U*_*avg*_	Average breakdown voltage
*σ*	Standard deviation
*w*	Pollution degree based on ESDD
*U*	Applied voltage on the insulator
*U*_*0*_	Cutting voltage
*n*	Cutting parameter
*k*, *β*, *c*	Parameters of Weibull distribution function
α, *k*_*w*_, *w*_50_	Parameters for breakdown voltage and failure probability versus pollution degree
*w*_*0*_	Cutting pollution degree
*U*_*c*_	Nominal voltage of the insulator
*h*_*0*_	Threshold of relative humidity percentage
*K*_*h*_	Constant related to the dry mode breakdown
*M*	Performance Metric
*M*_*0*_	Pre-event value of the performance metric
*M*_*pe*_	Post-event value of the performance metric
*M(t)*	Value of the performance metric at time *t*
*t*_*d*_	Starting time of degradation
*t*_*pe*_	Ending time of the incident
*tr*	Starting time of recovery actions
*tpr*	Completion time of the restoration phase
Δ*RI*_*k*_	Improvement of resiliency index upon implementing the *k*th solution
Δ*RI*_*max*_	Maximum amount of enhancement in the resiliency index
$C_{k}^{\;EUAC}$	Uniform annual cost of *the k*th solution
$C_{\max}^{\;EUAC}$	Maximum uniform annual cost of the suggested solutions

## 1 Introduction

Recently, climate changes and global warming have significantly increased the rate of high impact low probability (HILP) events [1,2]. HILP events have been causing serious challenges to power system sustainable operation worldwide, especially distribution systems [3]. Urmia Lake is an over-saturated Salt Lake located between East Azerbaijan and West Azerbaijan provinces in Iran. Urmia Lake was the largest in the Middle East and the sixth in the world at its original size before the drought [4]. A majority area of Urmia Lake has dried up due to the unbalanced in-out flow of water in the wake of the climate changes in recent years [5]. The residual salt layer in the dried part is risen and dispersed by the wind. It is then settled on electric insulators in nearby power distribution systems. The combination of salt contamination and humidity affects the performance of the distribution grid's insulators, remarkably. An insulator breakout and its surface flashover makes the protection systems remove the faulted section and leads to power network failure and cause negative social impact [6]. In the case of multiple breakouts in different insulators throughout the system, cascading outages may occur leading to a widespread blackout such as that took place in Khuzestan province, Iran, in February 2016 [7]. With distribution systems spread over large areas and using many insulators, along with increasing drought and drying lakes and rivers, analysing resilience against salt dust is now more crucial than ever.

**Fig. 1:**
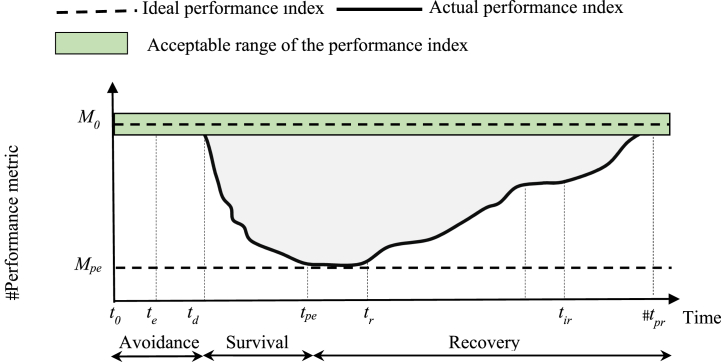
Resiliency curve of a feeder before, during, and after an HILP accident [9].

Resiliency is the ability of the system to predict and withstand against HILP events, confront their consequences in an anticipatory approach, and restore from the degraded situation [8,9]. Accordingly, the temporal response of the power system to an HILP event is divided into three phases: avoidance, survival, and recovery as shown in Fig. 1 [10,11]. In the avoidance phase, the system preparedness is enhanced by operation-oriented proactive measures to delay or smooth out the adverse impacts of the predicted event. In the survival phase, the operator aims to keep the system from total collapse by adopting appropriate corrective actions. Finally, the post-event restorative actions are taken to recover the system to the pre-event state.

Recently, several researches have been conducted on resiliency assessment and enhancement of power systems against natural disasters such as earthquakes [12], hurricanes [13], ice storms [14], and floods [15]. In Ref. [16], a two-stage long-term optimization framework is proposed for assessing and quantifying the energy system resilience against HILP created by. This paper proves that the vulnerability degree of energy systems against climate changes directly depends on the flexibility of the planner. In Ref. [17], a resilience oriented optimal energy management for a CCHP based microgrid is proposed. Minimizing the enhance resilience cost, minimizing operation and investment costs along with power loss reduction by feeder reconfiguration are the main goal of this paper. However, the type of HILP event and the fragility curves of the equipment are not modelled. In Ref. [18], resilient reconfiguration of distribution network feeders by optimal placement of on-line tie switches is proposed under different load scenarios. However, the resilience curves of feeders have not been reported. A similar approach is presented in Ref. [19] for optimal reconfiguration of distribution network in case of an accident, but the failure probability of equipment is not modelled according to the severity of the accident. In Ref. [20], the use of a fleet of electric vehicles to enhance the resilience of network against power outage is proposed using an agent-based modelling. However, the disaster type is not specified in this study. In Ref. [21], by determining the network topology change, an optimization approach is presented to reduce the power flow passing through the vulnerable lines of the network to reduce the probability of fire ignition. However, the improved resilience curve of network has not been reported.

Extreme dust storm can also be classified as an HILP natural disaster that may trigger simultaneous common-cause outages by reducing the insulation level of power system insulators. The frequency of dust storms has been increasing significantly in the West Asia and the Middle East due to climate changes in recent years [22]. Examples of the ruinous effects of extreme dust storms on the performance of the power system have been reported in Egypt, Saudi Arabia, Iraq, China, and Iran [23]. An extreme dust storm swept over the entire sub-transmission and distribution networks of Khuzestan province in Iran, on January 27, 2017, leading to a massive load shedding around 16.8 GWh for several consecutive days. Due to the widespread effects of extreme dust storms, the evaluation and enhancement of distribution system resiliency against this rare natural disaster looks as an outstanding practical requirement and an appealing research topic which is barely speculated so far. In Ref. [24], a new model based on stochastic mixed-integer linear programming model is presented for resiliency enhancement of electricity distribution networks against extreme dust storms. In this work, parameters such as load, air humidity, pollution level of insulators, line/substation damage, and repair time of components are all considered to be uncertain.

Reviewing the latest published works, it is found that no paper has yet evaluated and modelled the resiliency of the power system in face of extreme salt dust storms. This paper aims to fill the gap associated with resiliency analysis of distribution networks against salt dust. Due to the non-uniform distribution of dust particles on the power system insulators, it is quite complicated to evaluate the effect of dust deposition on the performance of electricity distribution networks. In this regard, leakage current and equivalent salt deposit density (ESDD) are commonly measured to monitor the performance of insulators in power systems [25]. In Ref. [26], a probabilistic approach is suggested to determine the chance of the arc propagation along the insulator surface on the ESDD intensity. To cope with the uncertainties, failure probability of the distribution grid insulators is calculated in Ref. [24] by comparing the voltage difference between two arbitrary points on the insulator surface and the electric strength of the air gap between the two points. This comparison is made for different levels of ESDD and humidity.

This paper presents a probabilistic method for resiliency assessment and improvement against salt dust and applies the proposed method on two real electricity distribution feeders in Iran. In this regard, an experimental approach is first developed for extracting fragility curves of insulators in terms of pollution degree and relative humidity. Fragility curves are then employed in a probabilistic optimal power flow (POPF) to obtain the system performance from the event onset till the completion of the restoration process. Having known the system performance from the supplied load perspective, new evaluation indices are introduced to assess the feeder resiliency. Next, various countermeasures are proposed as potential solutions to improve the resiliency indices of the feeders under study. The speculated solutions are then sorted based on a Benefit to Cost Ratio (BCR) index; and then per the available budget, a number of items with higher BCR index are adopted to be implemented.

The main contributions of this paper are listed as follows:

• Failure mechanism analyzing of insulators against the salt pollution and relative humidity using mathematical modelling and experimental analysis;
• Deriving 3-D fragility curves of insulators, illustrating the failure probability of insulators against the pollution degree and relative humidity;
• Proposing probabilistic method for resiliency assessment and improvement of distribution networks against salt dust;
• Identifying and prioritizing of Potential resiliency enhancement countermeasures are using the benefit to cost ratio (BCR) method.

The rest of the paper is organized as follows: Section 2 describes the procedure for extracting the fragility curves of insulators. Section 3 details the resiliency enhancement countermeasures for distribution systems against the salt dust. Section 4 presents the resiliency assessment and enhancement framework in addition to resiliency indices. Simulation results and sensitivity analysis are presented in Section 5. Finally, conclusive remarks are given in Section 6.

## 2 Proposed procedure for extracting fragility curves and failure analysis

In this section, the methods in the literature for extracting fragility curves of power system components are first reviewed. In the next step, based on the probabilistic process of the insulator breakdown, a novel method is proposed to extract the fragility curve of insulators as a joint function of pollution degree and relative humidity. Finally, an experimental procedure is developed to plot three-dimensional fragility curves of polymeric composite, glass, and porcelain insulators.

Different methods are proposed in the literature to extract the fragility curves of power system components: (i) statistical methods which scrutinize the historical data of similar events, (ii) experimental methods testing the device in question by controlled experiments, (iii) theoretical methods which analyse the mathematical and simulation models, (iv) consultative methods relied on expert judgments, and (v) combination of these methods [[27], [28], [29], [30], [31]]. In this research, experimental methods are utilized to extract the fragility curves of insulators due to their higher accuracy compared to other methods.

### 2.1 Probabilistic process of Insulator breakdown and failure

Insulators play a vital role in the normal operation of power systems. Insulators installed in electric transmission networks are usually made of porcelain, glass, or polymeric composites [32]. Porcelain and glass insulators have suitable electrical, mechanical, and thermal properties; however, they are vulnerable to pollution and humidity because of their hydrophilic surface [33]. On the other hand, polymeric insulators, especially insulators made of silicone rubber, have better electrical and mechanical properties. In addition, due to the great hydrophobicity surface of polymeric insulators, the humidity appears as discrete water drops. Therefore, extensive humid layers cannot be formed, which significantly lowers the probability of pollution flashover [34].

The pollution flashover is a probable mechanism for the breakdown of insulators. This mechanism is triggered on the surface of insulators by a conductive layer created by pollution and humidity [35,36]. In the first step, it is assumed that there is enough level of humidity required for triggering the breakdown. Therefore, the breakdown probability is a function of pollution degree on the surface of the insulator. In a probabilistic approach, the breakdown of equipment and related parameters are considered as random variables [37]. Usually, cumulative normal Gaussian distribution is used for the insulator's failure probability estimation against pollution as follows:

(1) $$P\left(U,w\right)=\frac{1}{\sqrt{2\pi }\sigma }\int_{0}^{U}\exp\left[-\frac{1}{2}{\left(\frac{u-U_{avg}\left(w\right)}{\sigma }\right)}^{2}\right]\;du$$

where u as the integral parameter is the breakdown voltage, U_avg_ and σ are average and standard deviation of the breakdown voltage, respectively. U_avg_ is deemed to be a function of pollution degree (w).

In the Gaussian distribution due to its symmetry, the probability of failure for low intensity of the incident is not zero, which is not the case according to the practical results. Experimental tests confirm that for the pollution levels less than a threshold value, pollution have zero adverse impacts on the piece of equipment under study [38]. Weibull distribution by an asymmetrical form fits better here [38]:

(2) $$P\left(U\right)=1-\exp\left[-{\left(\frac{U-U_{0}}{\beta U_{0}}\right)}^{k}\right]$$

where, parameters *U*_*0*_, *k*, and *β* are determined as follows [38]:

(3) $$U_{0}=U_{avg}-n\sigma$$

(4) $$k=\frac{1.38}{\ln\left(\frac{n}{n-1}\right)}$$

(5) $$\beta =\frac{n\times c}{\left(1-n\times c\right){\left(\ln\;2\right)}^{k}}$$

(6) $$c=\frac{\sigma }{U_{avg}}$$

in (2)–(6), *n* is the cutting parameter for Gaussian distribution at the cutting voltage (*U*_*0*_) which is equal to 4 based on IEC standards. For polluted insulators, it is about 2.5 according to experimental results [38,39].

The set of equations (1), (2), (3), (4), (5), (6) calculate the failure probability based on the breakdown voltage. To state the failure probability in terms of pollution degree, the relationship between the breakdown voltage and pollution degree can be used as follows [37]:

(7) $$U\left(w\right)=k_{w}\times w^{-\alpha }$$

where, parameters *α* and *k*_*w*_ can be obtained by experimental tests performed at the pollution degree of *w*, which is stated by ESDD [37]. In other word, Eqn (7) suggests conducting experimental tests to obtain the parameters required to relate the breakdown voltage to the pollution degree (ESDD, w). By substituting (7) in (2), the failure probability of insulators versus different pollution degrees can be achieved [39]:

(8) $$P\left(w\right)=1-\exp\left[-{\left\{\frac{1}{\beta }\left({\left(\frac{w}{w_{0}}\right)}^{\alpha }-1\right)\right\}}^{k}\right]$$

where *w*_*0*_ is mathematically expressed in (9) and defined as the cutting pollution degree in which the probability of failure for lower degrees of pollution is zero [39]:

(9) $$w_{0}=w_{50}{\left(1-n\times c\right)}^{1/\alpha }$$

where *w*_*50*_ corresponds to the pollution severity with a 50 % flashover probability. Substituting the nominal voltage of the insulator, *U*_*c*_, for applied voltage, *U* in (7), *w*_*50*_ can be calculated as [38]:

(10) $$w_{50}={\left(\frac{k_{w}}{U_{c}}\right)}^{1/\alpha }$$

The model described in (8) can be extended for representing the failure probability of insulators against joint pollution and humidity. According to Ref. [37], if the relative humidity percent is higher than the threshold value (*h*_*0*_), the breakdown voltage decreases exponentially versus relative humidity. To evaluate the simultaneous reduction of breakdown voltage against pollution and humidity, the following equation is suggested by Ref. [37]:

(11) $$U\left(w,h\right)=K_{h}\left[1-\frac{1}{1+\exp\left\{-\left(\frac{RH-h_{0}}{100}\right)\right\}}\right]+k_{w}\;w^{-\alpha }\left[\frac{1}{1+\exp\left\{-\left(\frac{RH-h_{0}}{100}\right)\right\}}\right]$$

where *RH* is the relative humidity percent, and *K*_*h*_ is a constant proportional to the breakdown voltage in the dry condition. In order to determine the fragility curve of insulators versus humidity and pollution, (11) should be substituted for *U*_*avg*_ in Weibull distribution equations (2), (3), (4), (5), (6). It should be noted that the nominal voltage is considered as the applied voltage, i.e. *U* = *U*_*c*_. Finally, equation (12) is proposed in this research to derive 3-D fragility curves of insulators, illustrating the failure probability of insulators as a joint function of humidity and pollution degree.

(12) $$P\left(w,h\right)=1-\exp\left[-{\left(\frac{1}{\beta }\left[\frac{Uc}{\left[K_{h}\left[1-\frac{1}{1+\exp\left\{-\left(\frac{RH-h0}{100}\right)\right\}}\right]+k_{w}\;w^{-\alpha }\left[\frac{1}{1+\exp\left\{-\left(\frac{RH-h0}{100}\right)\right\}}\right]\right]-n\delta }-1\right]\right)}^{k}\right]$$

### 2.2 Experimental steps to generate fragility curves

In this section, we focus on the proposed experimental method to determine the required parameters of the insulator's fragility curve versus pollution degree and relative humidity.

#### 2.2.1 Aging

In a given pollution degree and relative humidity, the breakdown voltage declines, and the failure probability grows by the insulator aging. In order to account for the aging effect, it is highly recommended to use naturally aged insulators installed in real distribution networks traversing through the questioned area or one with a rather similar condition. However, if such insulators aren't available, similar insulators should be artificially aged by the experiments according to IEC 61109 standard [40].

#### 2.2.2 Polluting

There are two main methods for applying artificial pollution on the surface of insulators: the salt fog test and the solid layer test [41]. In this research, similar to most studies, the solid layer test is adopted to pollute the outer surface of insulators due to its acceptable accuracy and fast implementation [42]. According to IEC 60507 standard, first, some pure water drops are sprayed on the surface of the insulator, and then kaolin powder is dumped on the water drops. The combination of the water layer and kaolin boosts the surface hydrophilicity of the insulator. As a result, polluting insulators with different severities becomes feasible. Finally, the pollution solution of lake salt (NaCl) is sprayed layer-by-layer on the surface of the insulator.

#### 2.2.3 Humidifying

According to IEC standards, there are three methods to generate different percentages of relative humidity: cold fog, steam fog, and mixed fog [42]. Since the impact of temperature on the pollution solution process and breakdown voltage is ignored, the cold fog method is suggested which is more consistent with the real situations in this study.

#### 2.2.4 Breakdown voltage test

The breakdown voltage is among crucial indices in the insulator's failure assessment. Hence, in the last step, the dielectric strength of the insulator is examined. In this regard, the power frequency voltage test is performed to determine the breakdown voltage in the various conditions of pollution and humidity. In this study, *σ* equals with 5 % which is a typical standard deviation of the breakdown voltage for experimental data [43]. The steps outlined in this section are being taken for insulators in various pollution degrees and relative humidity percentages to calculate the required parameters for plotting fragility curves.

### 2.3 Extracting fragility curves

**Fig. 2:**
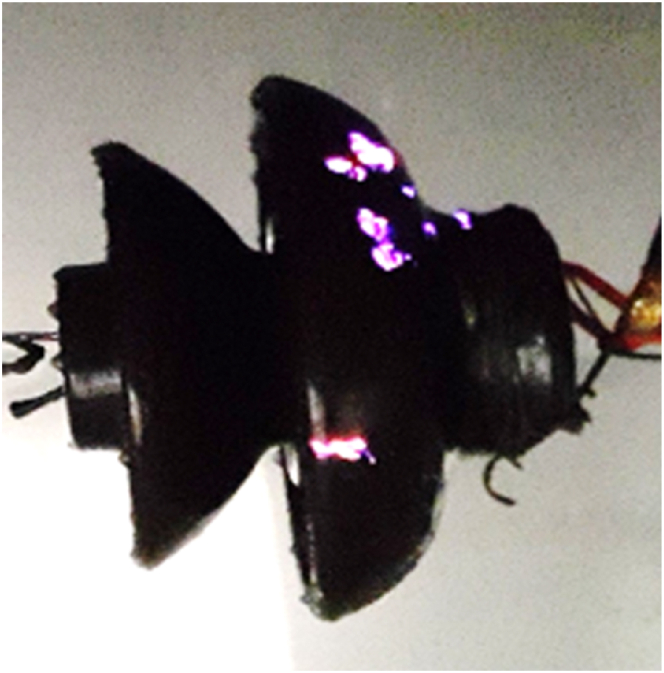
A sample of under-test porcelain insulators in the high-voltage laboratory.

In this section, experimental results are analysed to extract the 3-D fragility curves of insulators under study. Experimental tests have been performed in the high-voltage laboratory hosted in University of Tehran. In this research, polymer, glass, and porcelain insulator types are used which are currently employed in real conditions. Fig. 2 shows the under-test porcelain insulator.

**Fig. 3:**
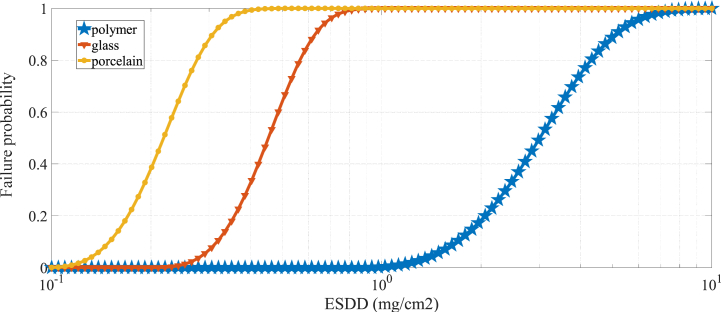
Fragility curves of insulators under study versus different degrees of pollution with utter humidity.

In these experiments, there is utter humidity (100 %) for insulator breakdown. Thus, the failure probability of insulator is analysed for different degrees of pollution. To establish the fragility curve for any particular humidity and pollution level, it is essential to accurately measure several key parameters for each insulator. Specifically, there are 8 critical parameters that need to be evaluated for each insulator. To ensure that these parameters are accurately determined, a minimum of 24 tests are required. However, to achieve a higher degree of accuracy and reliability in the obtained results, more than 50 tests are conducted. Having performed the experimental tests, the required parameters are determined as listed in Table 1 using (2), (3), (4), (5), (6), (7). Finally, by means of Weibull's probability function, the fragility curves of the studied insulators versus the pollution degree (denoted by ESDD) are obtained as plotted in Fig. 3. In summary, the steps related to extracting the fragility curve of insulators are as follows:

• Experimental tests: Conducting sufficient tests in the High Voltage Laboratory by varying salt pollution levels with ensuring adequate humidity to extract the required parameters listed in Table 1.
• Data Collection: Recording failure rates of different insulator types under test conditions.
• Weibull Probability Function: Applying the Weibull probability function (Eq. (8)) to model failure probabilities as a function of pollution degree.
• Fragility Curve Construction: Calculating and plotting the cumulative distribution functions (CDF) for each insulator type at various pollution levels.

**Table 1:** Required parameters for plotting fragility curves versus different degrees of pollution with utter humidity.

Insulator Type	k_w_	α	n	k	c	β
Polymer	13.53	0.37	2.5	2.7	0.0084	0.7155
Glass	5.60	0.6	2.5	2.7	0.0084	0.7155
Porcelain	3.78	0.57	2.5	2.7	0.0084	0.7155

Obviously, the polymeric composite insulator has the highest strength against the pollution. Porcelain insulators are more fragile in the same degree of pollution and glass insulators lie in between.

**Fig. 4:**
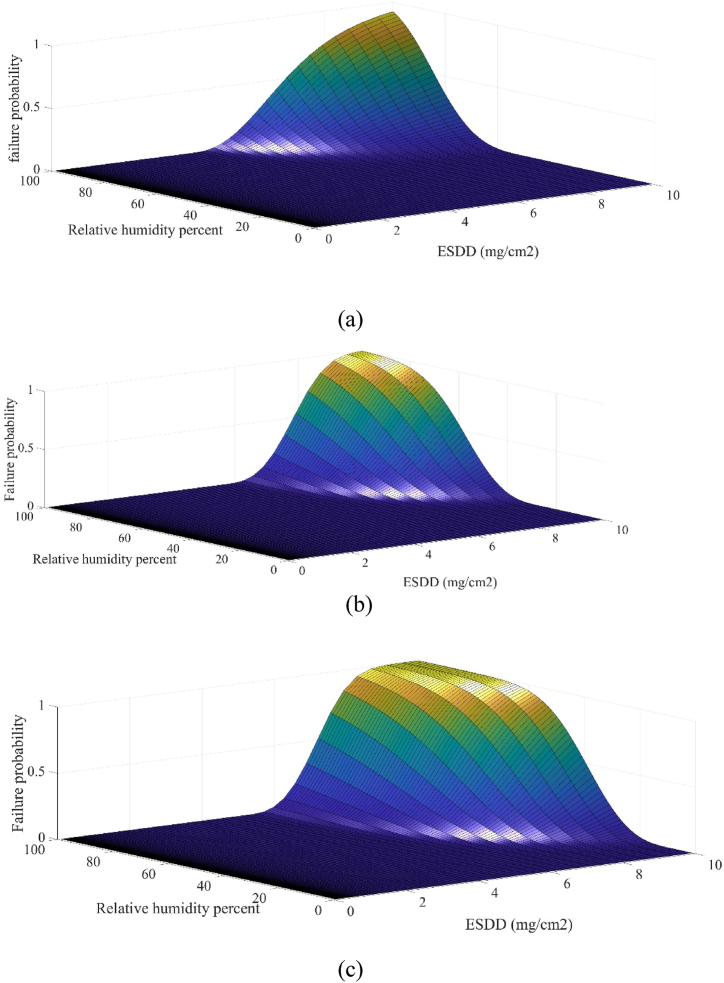
3-D fragility curves illustrating the failure probability of insulators as a joint function of humidity and pollution degree for (a) polymeric composite insulator, (b) glass insulator, and (c) porcelain insulator.

**Fig. 5:**
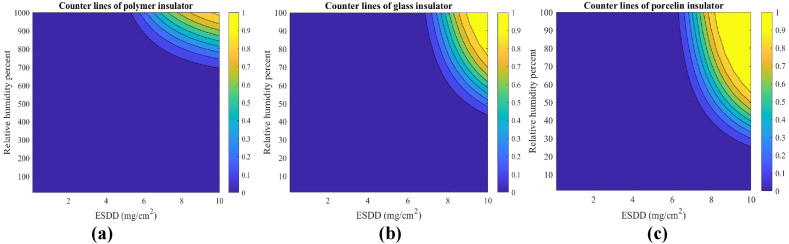
Counter lines of insulators according to the extracted 3-D curves for (a) polymeric composite insulator, (b) glass insulator, and (c) porcelain insulator.

**Fig. 6:**
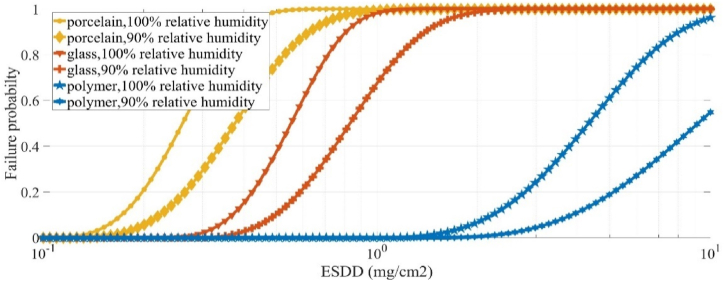
2-D fragility curves of the insulators under study for relative humidity levels of 90 % and 100 %**.**

Here, (12) is used to derive the simultaneous effect of relative humidity and pollution degree. To do so, the relative humidity effect is utilized on the insulators under study as stated in in Table 2. Substituting parameters of Table 1, Table 2 in (12), the 3-D fragility curves of the insulators are plotted in Fig. 4(a), (b), and (c). It is confirmed in Fig. 4 that the pollution has no adverse impact on the electric strength of the insulators in case of no humidity. Various studies, including those examining the performance of different types of insulators (such as porcelain, glass, and polymeric insulators), consistently show that in the absence of humidity, the electrical performance remains stable despite the presence of surface contaminants [44,45]. This is corroborated by experimental data indicating that without humidity, the insulator surface does not exhibit significant changes in electric field distribution or potential difference, thus preventing flashover [46]. To facilitate the understanding of Fig. 4, counter lines of insulators are plotted in Fig. 5 according to 3-D fragility curves shown in Fig. 4. It can be observed that by increasing relative humidity and ESDD, the failure probability is increased such that at ESDD = 10 and relative humidity percentage of 100, maximum failure probability is achieved. 2-D fragility curves are plotted in Fig. 6 for relative humidity levels of 90 % and 100 % to present a more detailed comparison between insulator types. According to Fig. 4, Fig. 6, the polymeric composite insulator is the strongest insulator type against humidity and pollution. Glass and porcelain insulators take the next ranks, respectively. In each degree of pollution, more relative humidity heightens the failure probability of the insulator. In addition, simultaneous growths of pollution and humidity results in a huge increment in the failure probability.

**Table 2:** Parameters for plotting fragility curves versus different levels of relative humidity.

Insulator type	K_h_	h_0_
Polymeric composite	70	70 %
Glass	64	70 %
porcelain	57	70 %

## 3 Resiliency enhancement countermeasures

In this section, based on expert knowledge and historical reports from similar incidents, a number of solutions are discussed to enhance the resiliency of the distribution grid under study against salt dust. Resilience enhancing countermeasures may be preventive, corrective, or restorative which are applied prior to, during, and after happening of HILP event, respectively [47]. In the following, a number of resiliency enhancement solutions against salt dust are presented in three category of preventive, corrective, and restorative approaches.

### 3.1 Preventive approaches

#### 3.1.1 Washing the insulators

Insulators are one of the main equipment of power systems and are responsible for insulating conductors with respect to the tower. Due to the existence of different pollution, insulating feature of the insulators is reduced over time [48]. Some of this pollution can be removed through washing [49]. So, washing is the most common way to improve the performance of an insulator against salt dust. In areas with a high level of pollution, insulators are washed regularly to keep their efficient performance. Therefore, washing the insulators is a primary solution to increase the resiliency of distribution systems against dust. IEEE has a standard for cleaning insulators which includes washing them when they are polluted [50]. In fact, washing insulators to improve their performance is not just an academic suggestion, but rather a common practice in industry.

#### 3.1.2 Implementation of underground cables

Although using underground cables is one of the most effective methods to decrease the impact of salt dust, it imposes high expenses on distribution systems. It could thus be implemented only in high-risk feeders.

#### 3.1.3 Using aerial cables

Aerial bundled cables have insulated phase conductors reinforced by central support conductors. The installation of such cables eliminates the risk of flash over which in turn decreases the vulnerability of insulators against salt dust.

#### 3.1.4 Removing dust sources

The origin of salt dust can be eliminated by avoiding salt erosion, improving the pasturages, strewing the gravel, and so on. The solution adopted for Owens Lake's dust challenges in the United States is an example of removing salt dust sources [51]. Due to extensive area of the distribution grid under study and heavy implementation costs, it is impossible to eliminate the origin of salt dust in Urmia Lake.

### 3.2 Corrective approaches

#### 3.2.1 Replacing the insulators

According to the fragility curves derived for different insulators in Section 2.3, the polymeric composite insulator has a better performance against the salt pollution and relative humidity than other types of insulators. Therefore, porcelain and glass insulators can be replaced with silicone rubber insulators to reduce the failure probability of pollution flashover.

#### 3.2.2 Using canopy for transformers

The use of canopy avoids the collection of salt pollution on the surface of transformer insulators and increases the creepage distance. Therefore, it is less likely to face with dry band arc and pollution flashover, leading to resiliency enhancement of distribution systems.

#### 3.2.3 Using pad-mounted substations

Pad-mounted and gas-insulated substations (GISs) are less affected by salt dust. However, it is too costly for distribution systems to replace air-insulated substations (AIS) with pad-mounted or GIS ones. Therefore, this solution may be utilized in regions with high degrees of pollution or those serving critical load points.

### 3.3 Restorative approaches

#### 3.3.1 Allocating fault detectors

The first step in the restoration stage is the detection of damaged distribution lines. The fault detector can help to locate defective sections quickly which in turn accelerates the recovery process.

#### 3.3.2 Expanding repair teams

Crew teams should be dispatched post event to recover the damaged lines based on the grid topology and loads priority. Therefore, increasing the number of crew teams expedites the service recovery. However, this solution comes with a huge fixed cost because of the increased number of personnel.

#### 3.3.3 Allocating distributed energy resources (DERs)

DERs can be employed to supply critical loads within isolated microgrids in emergency conditions imposed by the salt dust outages. Thus, optimal allocation of DERs in distribution systems enhances the resiliency of the distribution system [52].

#### 3.3.4 Extending maneuver points with adjacent feeders

In emergency conditions, distribution system operator (DSO) can switch with adjacent feeders to transfer critical loads of the damaged feeder to a healthy feeder via a manoeuvre point. In addition, the manoeuvring capability enhances the efficiency of feeder restoration. Therefore, allocating the manoeuvre points in optimal locations can improve the distribution system resiliency.

#### 3.3.5 Installing remote-control switches (RCSs)

Relying on emerging automation systems and advanced communication technologies, modern situational awareness of distribution system has been turning into a viable option. By the aid of RCSs, DSO rapidly isolates the damaged sections of the system so that repair teams can safely start working to recover the system. In addition, the distribution system can be reconfigured optimally using RCSs to transfer loads to normal adjacent feeders via manoeuvre points.

#### 3.3.6 Extending manual disconnecting switches

Disconnecting switches with a manual mechanism such as cut-out fuses and aerial disconnectors can also introduce advantages mentioned for RCSs. These switches are more affordable compared to RCSs.

## 4 Resiliency assessment framework

In this section, the resilience curve modelling process is first presented and some indices for quantifying the resilience is introduced. Then, an index for cost-benefit analysis of resilience improvement solutions is proposed. Finally, the proposed steps for evaluating the resiliency enhancement solutions are presented.

### 4.1 Resiliency curve modeling and introducing resilience assessment indices

**Fig. 7:**
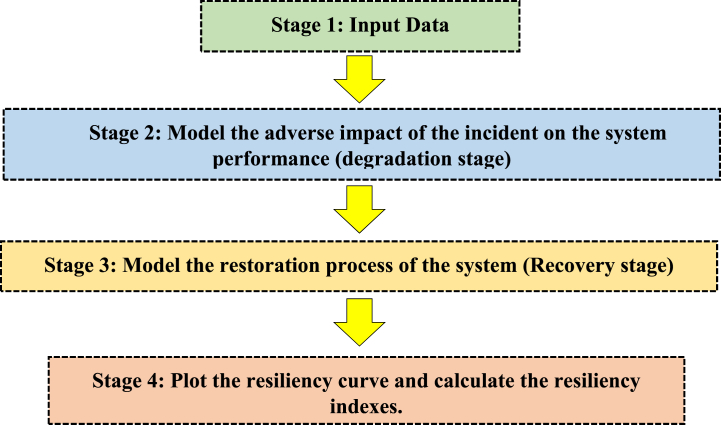
Proposed framework for modelling the resiliency curve of distribution feeder.

The proposed framework for modelling the resiliency curve of distribution feeder is presented in Fig. 7, which can be summarized in four stages. First stage is the required data preparation. In the next two steps, the degradation and recovery stages are modelled which makes it possible to calculate resiliency indices in the fourth stage. Input data includes: (i) certain sections of the distribution system vulnerable to salt dust, (ii) fragility curves of vulnerable insulators, (iii) geographical and electrical structure of the distribution system under study, (iv) load data, (v) manoeuvre points with adjacent feeders, (vi) data related to DERs, and (vii) the number of repair crew teams.

**Fig. 8:**
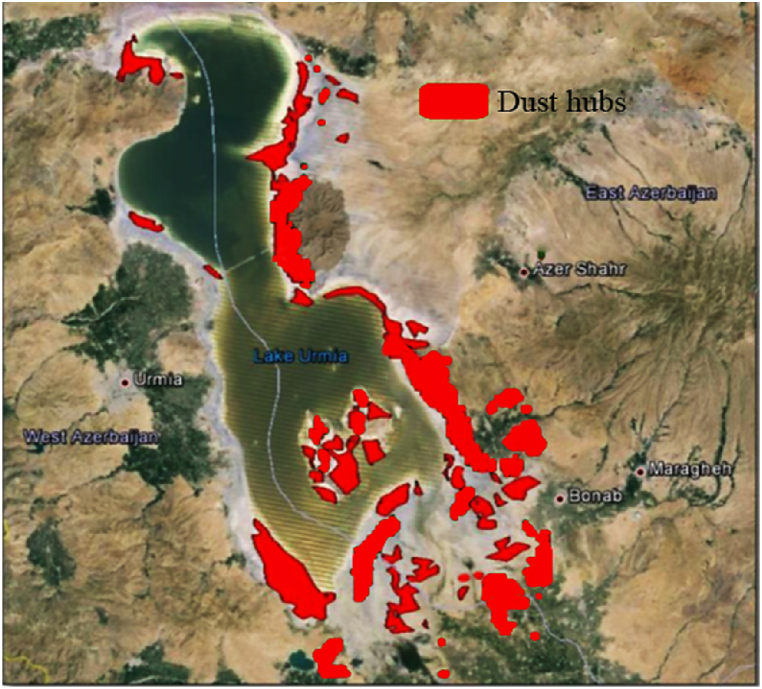
Salt dust hubs around Lake Urmia [53].

Vulnerable sections of the distribution system are identified based on the dust chasms of the area under study. Lake Urmia, formerly one of the largest saltwater lakes in the world, is now in danger of drying up and being destroyed. This issue serves as a warning for the formation of active salt dust hubs in the region around Lake Urmia. Remote measurement technology, which monitors suspended particles in the atmosphere via optical or thermal techniques, is commonly used to identify potential dust-rising hotspots. In this research, Aerosol Optical Depth (AOD) is employed for this purpose. This method considers the intensity and frequency of dust occurrence to determine and prioritize the areas with high salt dust generation potential. The salt dust hubs around Lake Urmia are depicted in Fig. 8.

According to Fig. 8, most of the dust hubs are located on the eastern shore of Lake Urmia. The dried areas caused by the lake retreat in the southern part are also among the areas with high potential for dust generation. In addition, an area in the northwest of the lake, two sites on the western shore of the lake, and an area in the southwestern part have also been identified as hotspots for salt dust events. Notable reasons for creating basins of salt dust are the intermittent drying and wetting of the soil. In addition, barren and saline lands are more prone to create salt dust hubs. Following identifying the salt dust hubs, the GIS map of the distribution grid should be analysed to determine the exposed areas to salt dust. In this research, Shourkand and Miandouab feeders were chosen as high-risk feeders to salt dust.

In order to extract the temporal performance of the system in the degradation and recovery stages, a sequential Monte Carlo method is employed to capture the stochastic behaviour of insulator outages. Given specified values of pollution (ESDD) and relative humidity, an insulator might fail by a flashover or continue its normal operation. In order to capture the stochastic behaviour of insulator outages, the outage probability of each insulator (taken from its associated fragility curve) is compared with a uniformly distributed random number, r∼U(0,1), to determine whether the associated branch is damaged or still in service. Having updated the network topology, an OPF is conducted to find out the amount of load supplied in the distribution system under study. The probability OPF (POPF) problem is formulated to determine the optimal operating conditions of feeder [54,55]. The primary goal of OPF is to maximize the load supplied to the distribution system [56]. This optimization ensures that the network operates efficiently even under the altered conditions caused by insulator outages. The Mont Carlo simulation is used to run the depicted process for a large number of iterations at each time slot to preserve the accuracy. Each iteration accounts for different random samples of insulator outages, capturing the stochastic nature of the problem. The supplied load calculated by POPF in each iteration is recorded, providing a series of outcomes that reflect the variability of the system under probabilistic conditions. Finally, the supplied load of the system at each time slot is obtained by averaging outcomes of all iterations. This averaging process helps to smooth out the variability and provides a robust estimate of the supplied load under probabilistic conditions. Repeating the similar iterative OPF for all time slots, the resilience curve is obtained from the supplied load viewpoint. Thus, the supplied load is considered as the performance metric of the system temporal response to the salt dust. In other word, the supplied load is considered as the performance metric of resilience curve shown in Fig. 1.

Having obtained the temporal response of the system, resiliency indices should be calculated to provide an assessment indicator for the system resiliency. In this research, VI (vulnerability index), DI (Degradation index), REI (restoration efficiency index), and RI (resiliency index) are used to assess the system resiliency [15,57]. The VI index represents the vulnerability of the studied feeder against a destructive event. Consider the performance metric (*M*) depicting the temporal response of the system in Fig. 1. The *VI* index is defined as the static drop of performance metric compared to its normal value before the incident (regardless of the path taken in the degradation phase) as follows:

(13) $$VI=\frac{M_{o}-M_{pe}}{M_{o}}$$

where *M*_*0*_ is the pre-event value of the performance metric (supplied load), and *M*_*pe*_ is the post-event value of the performance metric. According to (13), the ideal value for *VI* is zero, indicating that the event has no impact on the feeder's performance while its value in the worst condition is one, which means that the feeder is entirely un-functional. As the immediate drop of the system performance at the event onset is more sever, VI will take higher values.

The *VI* index gives a static view of the degradation phase but does not reflect the temporal behavior of the system in this phase. To cope with this issue, the *DI* index is utilized:

(14) $$DI=\frac{\int_{t_{d}}^{t_{pe}}\left(M_{o}-M\left(t\right)\right)dt}{M_{o}\left(t_{pe}-t_{d}\right)}$$

where *t*_*d*_ is the start time of degradation, and *t*_*pe*_ is the end time of the incident. *M(t)* is the value of the performance metric at time *t*. The value of *DI* is zero in the ideal condition and one in the worst-case scenario (i.e., complete, and rapid fall of the system). DI index measures the drop of the resilience curve considering the path taken in the degradation phase. Thus, it is interpreted as a dynamic metric of resilience. It is worth noting that the assessment of the degradation phase should consider both *VI* and *DI* indices to account for the static and dynamic aspects of the degradation phase.

To evaluate the efficiency of the restoration stage, the *REI* index is used as follows:

(15) $$REI=\frac{\int_{t_{r}}^{t_{\mathrm{pr}}}\left(M\left(t\right)-M_{pe}\right)dt}{\left(M_{o}-M_{pe}\right)\left(t_{\mathrm{pr}}-t_{r}\right)}$$

where *t*_*r*_ and *t*_*pr*_ are initial and completion times of the recovery stage, respectively. The ideal value of this index is one, which means the restoration process is done right away after degradation while in the worst case of no restoration, it becomes zero. A quick recovery to the initial operation point entails a higher value of REI. According to equation (15), REI is the proportion of the area under the restoration path to the total area under the nominal (initial) operation line within the restoration phase.

The mentioned indices assess the system performance in degradation and recovery stages. In order to have an overall view of resiliency, the whole resiliency curve should be accommodated to generate a separate index, that is *RI* index:

(16) $$RI=\frac{\int_{t_{d}}^{t_{\mathrm{pr}}}M\left(t\right)dt}{M_{o}\left(t_{\mathrm{pr}}-t_{d}\right)}$$

The value of *RI* is between zero and one. A higher value for this index indicates a more resilient system against the HILP event. So, RI can be employed to numerically judge the resilience curve from the event onset to the end of the restoration phase. According to equation (16), RI is the proportion of the area under the resilience curve to the total area under the nominal (initial) operation line from the event onset to the end of the restoration. Despite most of the resiliency indices in the literature, the indices employed in this study are normalized (between zero and one). Therefore, they can be utilized to compare different distribution systems fairly. Besides, unlike static reliability metrics, the proposed resiliency metrics measure the system's temporal response to the incident.

### 4.2 Cost-benefit analysis (BCR index)

It is obvious that implementation of all suggested solutions has the greatest impact on distribution system resiliency. However, due to the limited budget available for resiliency enhancement of distribution systems, it is impossible to implement all solutions. Therefore, the *BCR* index is employed in this research to select the most efficient solutions for enhancing the resiliency of the distribution system under study. Despite the use of BCR in other research fields [58], the idea of BCR for prioritizing resilience enhancement options is a contribution to the literature. The use of BCR in this work removes the need for optimization methods with heavy computational burden which is a serious concern in resilience studies. For each solution *k*, the *BCR* index can be calculated as the ratio of normalized benefit to normalized cost as follows:

(17) $$BCR_{k}=\frac{\Delta RI_{k}/\Delta RI_{\max}}{C_{k}^{\;EUAC}/C_{\max}^{\;EUAC}}$$

where Δ*RI*_*k*_ is the resiliency improvement resulting from the implementation of solution *k* and Δ*RI*_*max*_ is the highest amount of enhancement in the resiliency. In addition, $C_{k}^{\;EUAC}$ is the uniform annual cost of solution *k* and $C_{\max}^{\;EUAC}$ is the maximum uniform annual cost of the suggested solutions.

### 4.3 Proposed framework for evaluating the resiliency enhancement solutions

Considering the preventive, corrective and restorative approaches presented in section 3 to enhance the resilience of the distribution network against salt dusts, the following steps are proposed for evaluating the resiliency enhancement solutions:

1) Determining the resiliency enhancement solutions of distribution network against salt dusts: in this step some practical preventive, corrective and restorative approaches are defined and listed for improvement of resiliency.
2) Assessing the network resiliency in the current condition and calculate resiliency indices: In this step, the network is first evaluated under normal operating conditions and the base resiliency curve is extracted. This initial evaluation provided a baseline understanding of the network's performance.
3) Assessing the network resiliency after implementing each solution: in this step, improved resiliency indices are calculated and new resiliency curve is plotted to evaluate the impact of the impact of each corrective, preventive or restorative solution.
4) Ranking the potential solutions based on BCR index: The budget required for the implementation of each method should be considered. By assessing both the effectiveness and cost of the enhancement measures, the most efficient strategies for improving network resilience within the available budget constraints are prioritized and selected.

## 5 Results and discussions

In this section, the proposed resiliency enhancement methodology is implemented on two high-risk feeders in the west Azerbaijan power distribution grid, namely Shourkand and Miandouab feeders. In the first subsection, these two feeders are first introduced, and then in the second subsection, the resilience indices for the base state and then for the implementation of resilience improvement solutions are extracted and the resilience curve is plotted for each solution. In the third subsection, the improvement of the resilience curve due to the simultaneous implementation of several solutions is discussed, and finally, in the fourth subsection, the obtained results are discussed.

### 5.1 Introducing two high-risk feeders of Shourkand & Miandouab

**Fig. 9:**
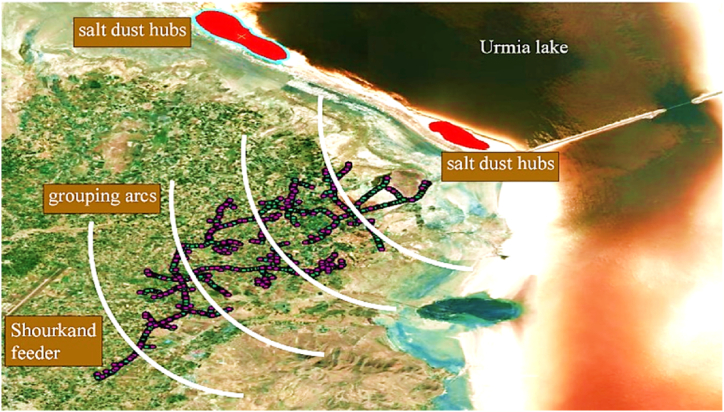
Aerial view of geographical topology of Shourkand feeder, dust hubs, and the insulator groups.

In this section the features of two high-risk feeders of Shourkand & Miandouab are presented. Fig. 9 shows an aerial photography of Shourkand feeder, which is a long medium voltage (MV) feeder routed in west of Urmia Lake. The vicinity of the feeder to Urmia Lake and salt dust hubs makes it highly vulnerable to pollution flashover. Most insulators installed in West Azerbaijan are made of porcelain which is highly vulnerable to pollution flashover. It is worth mentioning that all MV lines in Shourkand feeder are executed by aerial conductors. In order to capture the diversity of hazard levels, insulators are categorized based on their types and exposed degree of pollution. To do so, the distance between the insulator and the dust hub is used as a criterion for classifying the insulators into separate groups. In this study, the prevailing wind direction is utilized to determine the direction of salt dust movement. Considering the effective dust basin for the distribution system, arcs in Fig. 9 demonstrate the uniform distance from the dust basin.

**Fig. 10:**
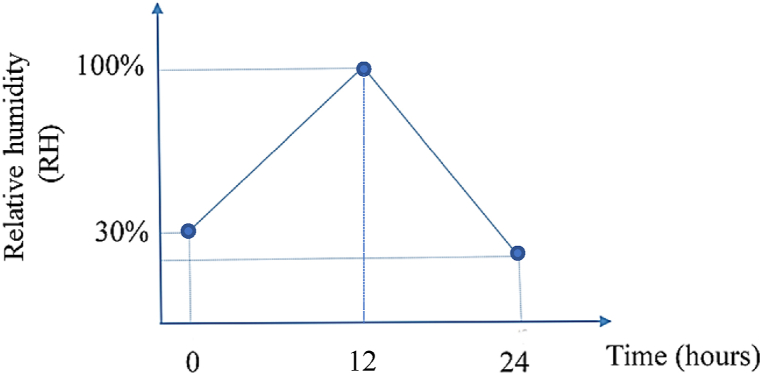
Time-variant relative humidity for the day under study in Shourkand feeder.

As discussed previously, the pollution flashover is a function of pollution degree and relative humidity. For pollution degree, the natural pollution degree is utilized for numerical analysis; in other words, the site insulator is used in the laboratory to extract the fragility curves. For humidity, the time-variant relative humidity for the day under study is illustrated in Fig. 10 based on historical data received from the local weather agency. Fig. 10 shows that the percentage of relative humidity around the Shourkand feeder at 12 midnight is about 30 %, while its amount increases to 100 % by 12 noon. After 12 noon, the percentage of relative humidity decreases again until 12 midnight.

**Fig. 11:**
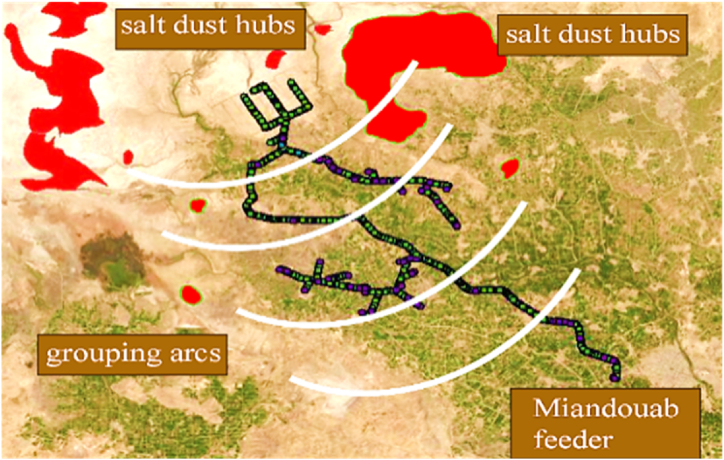
Geographical topology of Miandouab feeder, dust hubs, and the insulator's grouping arcs.

**Fig. 12:**
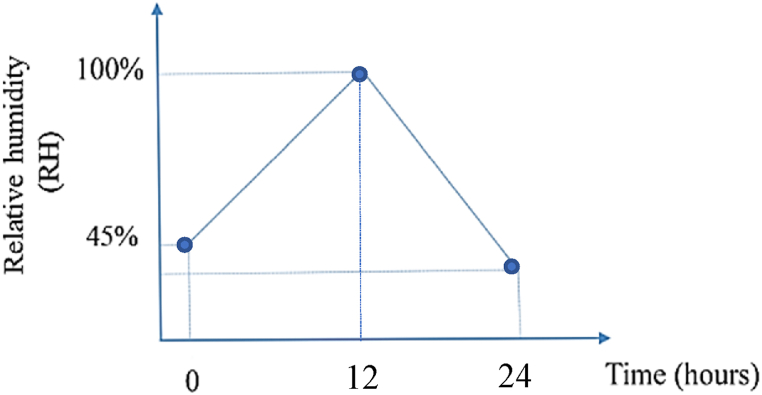
Variations of relative humidity percentage in one day in Miandouab feeder.

Miandouab feeder is another vulnerable feeder which is located in south of Urmia Lake. Fig. 11 shows the geographical topology of Miandouab feeder in the GIS environment, nearby salt dust hubs by red areas, and feeder's insulators grouping arcs. Since this feeder is near many salt dust hubs and most of the insulators are made of porcelain and glass, it is very vulnerable to salt dust. Besides, Fig. 12 demonstrates the maximum relative humidity variations in one day. This figure is based on the data from meteorological centres in the Miandouab feeder's region. Fig. 12 shows that the percentage of relative humidity at its lowest level (at 12 midnight) in the Miandouab feeder is about 45 %, which is more than the lowest relative humidity reported around the Shourkand feeder.

### 5.2 Simulation and extraction of resilience assessment indices and resilience curves

#### 5.2.1 Resilience assessment of Shourkand feeder

Two crew teams are considered for the Shourkand feeder while no emergency DERs are available in this feeder. Shourkand feeder has five manoeuvre points with adjacent feeders. It should be noted that when the salt dust occurs, all the feeders in the region are damaged at different levels. Thus, the availability of the adjacent feeders is not guaranteed. The probability of manoeuvre points being available is considered 0.5 in the sequential Monte Carlo simulations. Since Shourkand feeder is routed in a rural region, the loads are homogeneous and have the same level of supply priority for the distribution grid. Due to the lack of detailed data on load values in this feeder, the capacity rates of distribution transformers are used to estimate load point demands. To this end, the supplied load of Shourkand feeder is distributed based on the capacity ratios of transformers. Table 3 details proposed resiliency enhancement solutions along with associated costs, based on the advice given by experts from west Azerbaijan electricity distribution company. These solutions will further be ranked for possible implementation in practice.

**Table 3:** Proposed resiliency enhancement solutions along with associated costs.

Solution #	Solution description	Cost (Million Rials)
1	Allocating 8 fault detectors along the feeder	250
2	Washing the polluted insulators	210
3	Replacing insulators (400 insulator)	313
4	Adding another crew team	2160
5	Using emergency DERs (back-up diesel generators)	2000
6	Using canopy for transformers	120
7	Implementation of underground cables in high-risk regions	38810
8	Extending manoeuvre points with adjacent feeders	4000
9	Installing 3 RCSs	1200
10	Replacing 10 aerial distribution substations with pad-mounted ones	6000
11	Using aerial bundled cables	7500
12	Installing 6 cut-out fuses	60

**Fig. 13:**
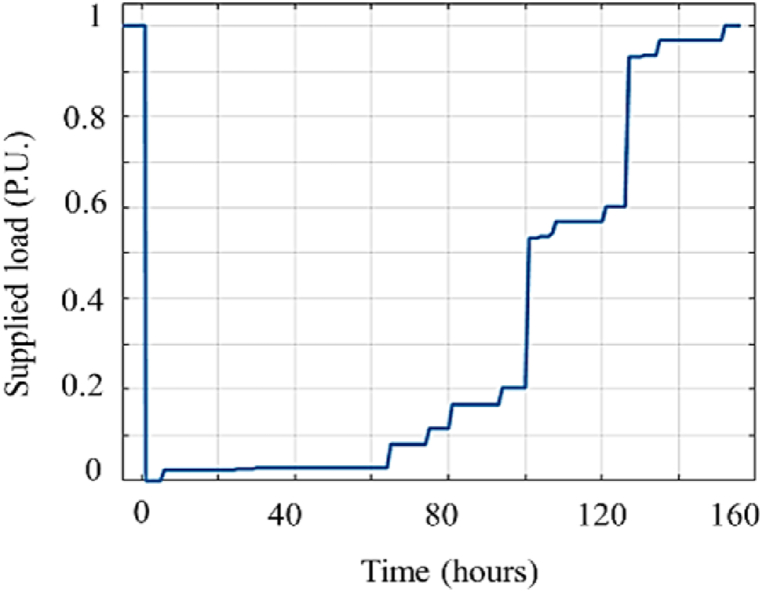
The resiliency curve of Shourkand feeder with no enhancement measures (base case).

The feeder resiliency is initially assessed in the current base case where no resiliency enhancement measures are implemented. Fig. 13 shows the resiliency curve of Shourkand feeder in the base case. Accordingly, the feeder supplies the entire load prior to the salt dust-originated incident. At the incident onset, the supplied load is dramatically reduced due to huge number of insulator breakouts, radial structure of the feeder, existence of long branches lacking enough isolating switches, and high vulnerability of installed insulators to salt pollution and relative humidity. The recovery actions are taken to restore the supplied load to its pre-event level (i.e., 1 p.u.) in 155 h from the initiation of the incident. Besides, the resiliency metrics of the feeder in base case, are reported in Table 4. Accordingly, both *VI* and *DI* are equal to one, which means the feeder is completely vulnerable to salt dust events.

**Table 4:** Resiliency assessment metrics for Shourkand feeder for each implemented solution.

Item	*VI*	*DI*	*REI*	*RI*
Base case	1	1	0.22	0.25
Solution 1	1	1	0.25	0.27
Solution 2	1	1	0.46	0.49
Solution 3	1	1	0.34	0.37
Solution 4	1	1	0.26	0.27
Solution 5	1	1	0.42	0.45
Solution 6	1	1	0.23	0.27
Solution 7	1	1	0.22	0.26
Solution 8	1	1	0.37	0.36
Solution 9	1	1	0.27	0.30
Solution 10	1	1	0.49	0.52
Solution 11	1	1	0.26	0.27
Solution 12	1	1	0.43	0.44

Fig. 13 represents the expected load supplied from the event onset to the end of the restoration process. To extract this figure, a probabilistic optimal power flow (POPF) is employed to obtain the supplied load at each time slot. The uncertainty of the proposed model is originated from the probabilistic vulnerability process of the components which is depicted by associated fragility curves. Given specified values of pollution (ESDD) and relative humidity, an insulator might fail by a flashover or continue its normal operation. In order to capture the stochastic behavior of insulator outages, the outage probability of each insulator is compared with a uniformly distributed random number to determine whether the associated branch is damaged or still in service. Having updated the network topology, an OPF is conducted to find out the amount of load supplied in the distribution system under study. The Mont Carlo simulation is used to run the depicted process for a large number of iterations at each time slot to preserve accuracy. Finally, the supplied load of the system at each time slot is obtained by averaging outcomes of all iterations (e.g., Fig. 13). Given the supplied load resilience curve, assessment indices (VI, DI, REI, and RI) can be calculated. Regarding Fig. 13, some key points are considered in the proposed model and summarized as follows:

• Following the incident, due to the radial nature of the network, damage to the first pole of the distribution network (caused by the breakdown of the associated insulator) will cause the supplied load to approach zero quickly.
• It takes time to dispatch the repair teams to restore the network. In addition, emergency generators cannot be operated until the repair teams confirm that the reconfigured topology of the network is secured. Thus, most of the load points remain unsupplied for some hours.
• As seen in Fig. 13, supplied load is enhanced gradually as repair teams restore the damaged branches, restored branches are isolated from damaged branches and connected to the upstream network, and emergency generators start operating. Finally, the supplied load returns to its initial level as the recovery process is fulfilled.

**Fig. 14:**
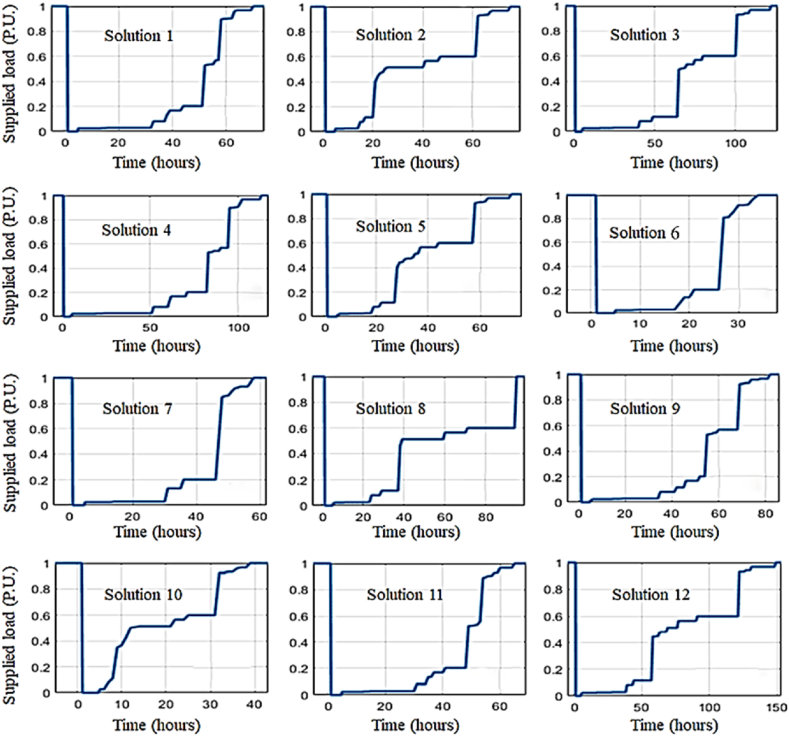
Resiliency curves of Shourkand feeder upon implementing enhancement solutions.

The resiliency curve and metrics of Shourkand are presented in Fig. 14 and Table 4 having applied resiliency enhancement solutions given in Table 3. In this paper, 12 solutions are presented to enhance the system's resilience. As illustrated in Fig. 14, each solution enhances the resiliency curve at least in one of the three phases: avoidance, survival, or restoration. Accordingly, the resiliency assessment metrics are partially improved for each solution as inferred from Table 4. The effect of a new solution is observed on the supplied load, and a new resilience curve is derived accordingly, with relevant indices calculated as a result. Each of these solutions is not necessarily associated with the adoption of a new fragility curve. Only some of the proposed solutions require the adoption of new fragility curves, while others mainly affect the system structure. For example, washing polluted insulators, replacing insulators, and replacing aerial distribution substations with pad-mounted ones necessitate new fragility curves. Historical data of similar components and expert knowledge are used to fit these new fragility curves. Conversely, other solutions, such as the installation of a fault detector or cutout, do not affect the system's strength and only aid in the restoration process, thus not requiring new failure curves. By incorporating these solutions, the network structure in MATLAB software is altered, impacting power flow, resilience, and indices. Additionally, adding a crew team generally reduces recovery time without changing the fragility curves of the poles. Furthermore, a new failure curve is determined for transformers with canopy using data from similar components in adjacent networks, based on historical data and expert knowledge. Each solution either introduces a new fragility curve (such as replacing insulators) or reconfigures the network topology (such as installing cut-out fuses). The same Monte Carlo-based OPF is performed once a given solution is implemented, and the resilience curve/metrics are obtained accordingly. For instance, if insulators are replaced with stronger ones, the outage probability of insulators can be taken from new fragility curves and compared with the uniformly distributed random number to determine whether the associated branch is damaged or remains in service. The iterative OPF process is then used to extract the supplied load resilience curve. Installation of cut-out fuses in the distribution system does not alter the fragility curves of system components but provides additional flexibility to isolate faulted lines and reconfigure the network topology. Therefore, the initial part of the resilience assessment process (determining whether the associated branch is damaged or remains in service) is performed similarly. However, the iterative OPF is applied to the distribution system with the added cut-out fuses. The impact of adding cut-out fuses is then reflected in the power flow results, supplied load resilience curves, and resilience indices, respectively.

Due to budget limitation, it's often impossible to implement all resiliency enhancement solutions proposed in Table 3. To cope with this challenge, suggested solutions are prioritized in Table 5 based on *BCR* index. According to this table, adding six cut-out fuses is the most efficient solution for resiliencye enhancement of Shourkand feeder.

**Table 5:** Prioritized resiliency enhancement solutions for Shourkand feeder based on *BCR* index.

Solution #	Description	BCR
12	Installing 6 cut-out fuses	316.23
3	Replacing insulators (400 insulator)	61.997
2	Washing the polluted insulators	55.886
6	Using canopy for transformers	53.903
1	Allocating 8 fault detectors in distribution system	25.873
9	Installing 3 RCSs	8.6244
10	Replacing 10 aerial distribution substations with pad-mounted ones	6.4683
11	Using aerial bundled cables	5.1747
8	Extending manoeuvre points with adjacent feeders	4.5278
4	Adding another crew team	2.9946
5	Using emergency DERs (back-up diesel generators)	1.4877
7	Implementation of underground cables in high-risk regions	0.066

#### 5.2.2 Resilience assessment of Miandouab feeder

Similar to the Shourkand feeder, all MV lines are overhead (aerial conductors) and the feeder does not have any emergency DERs. Two repair crew teams are assigned to this feeder. Miandouab feeder has two manoeuvre points with adjacent feeders. To incorporate the vulnerability of adjacent feeders against salt dust events, the probability of the manoeuvre point's functionality is considered 0.5 in the sequential Monte Carlo simulations. Besides, this feeder is developed in the rural region and the loads have the same level of importance for the distribution grid. Due to the lack of loading data, the capacity ratios of the distribution transformers are used to distribute the supplied load of the Miandouab feeder in the resiliency studies. It should be noted that the total supplied load is converted into per unite value (i.e., 1 p.u.).

**Fig. 15:**
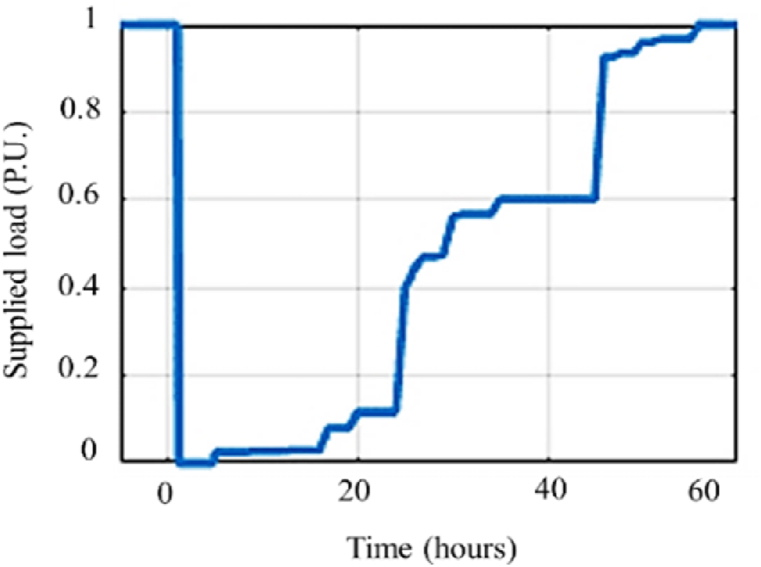
Resiliency curve of Miandouab feeder in the base case without implementing any solution.

Fig. 15 shows the resiliency curve of Miandouab feeder in the base case, in which no resiliency enhancement action is adopted. Accordingly, the feeder supplies all of the loads (i.e., 1 p.u.) before the incident. At the incident onset, supplied load is reduced significantly. The recovery process restores the interrupted loads completely after 60 h. In addition, resiliency indices of Miandouab feeder in the base case are listed in Table 6. It is inferred that similar to Shourkand feeder, Miandouab feeder is vulnerable to salt dust and relative humidity (*VI* = 1, *DI* = 1), and the resiliency index is 0.3 which is far below than the ideal condition (*RI* = 1).

**Table 6:** Resiliency assessment metrics for Miandouab feeder.

Solution number	*VI*	*DI*	*REI*	*RI*
Base case	1	1	0.22	0.30
1	1	1	0.28	0.42
2	1	1	0.51	0.37
3	1	1	0.38	0.31
4	1	1	0.27	0.34
5	1	1	0.47	0.31
6	1	1	0.29	0.35
7	1	1	0.25	0.32
8	1	1	0.38	0.32
9	1	1	0.31	0.36
10	1	1	0.57	0.35
11	1	1	0.28	0.42
12	1	1	0.45	0.57

**Fig. 16:**
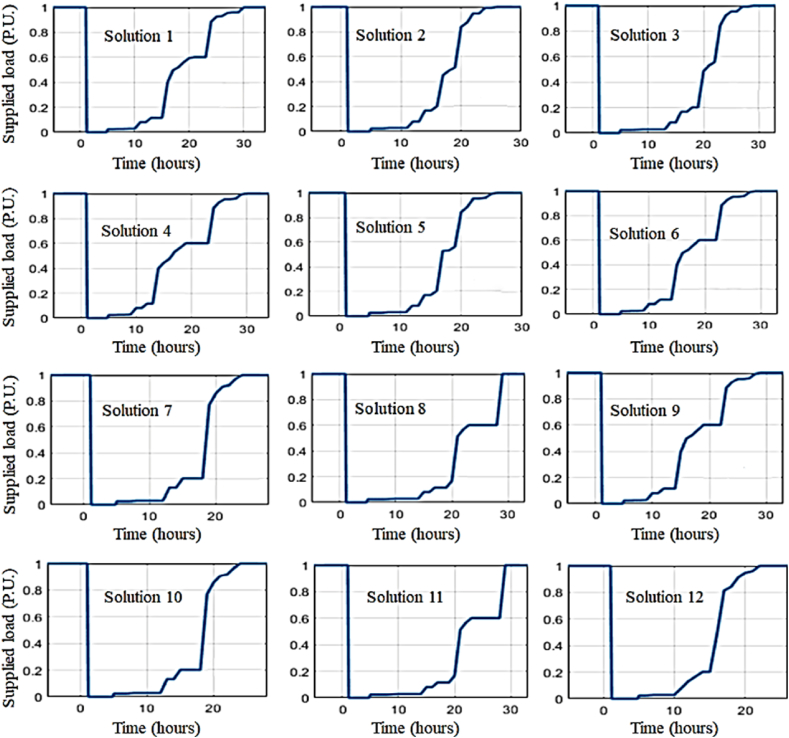
Resiliency curves of Miandouab feeder upon implementing enhancement solutions.

Fig. 16 compares the resiliency curves of Miandouab feeder upon adopting enhancement measures. In addition, Table 6 reports the resiliency indices in the base and reinforced cases. Each reinforcement solution improves at least one of the resiliency indices. However, Table 7 prioritizes the proposed solutions based on *BCR* index. Similar to Shourkand feeder, adding six cut-out fuses is the most efficient solution for enhancing the resiliency of Miandouab feeder.

**Table 7:** Prioritized resiliency enhancement solutions for Miandouab feeder based on *BCR* index.

Solution #	Description	BCR
12	Installing 6 cut-out fuses	431.22
6	Using canopy for transformers	136.18
1	Allocating 8 fault detectors in distribution system	93.961
3	Replacing insulators (400 insulator)	36.156
2	Washing the polluted insulators	29.414
11	Using aerial bundled cables	18.792
9	Installing 3 RCSs	14.469
4	Adding another crew team	7.0925
10	Replacing 10 aerial distribution substations with pad-mounted ones	2.7235
8	Extending manoeuvre points with adjacent feeders	0.7659
5	Using emergency DERs (back-up diesel generators)	0.6128
7	Implementation of underground cables in high-risk regions	0.3421

### 5.3 Simultaneous implementation of several solutions to improve the resilience

**Fig. 17:**
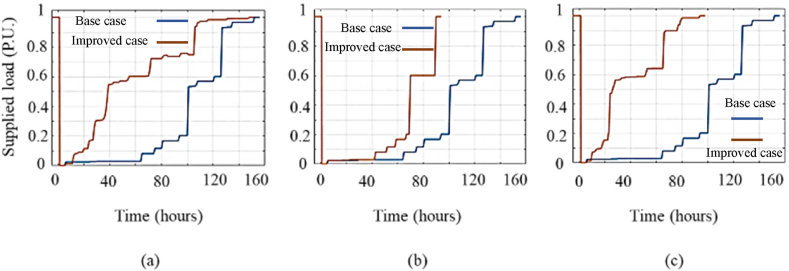
Illustrative comparison of Shourkand feeder resiliency curve for the base case and (a) implementation of solutions 12 and 3, (b) implementation of solutions 12, 3, and 6, and (c) implementation of all solutions.

In this section, to aggregate the individual impact of resiliency enhancement solutions presented in the previous sections, several solutions are adopted simultaneously and the obtained results are discussed. Fig. 14 and Table 4 illustrate that implementing a single solution do not increase the resiliency level of Shourkand feeder, sufficiently. Hence, the most efficient solutions should be taken simultaneously considering the budget limitation. Fig. 17 compares the resiliency curve of Shourkand feeder in the base case along with cases: (a) simultaneous implementation of solutions 12 and 3 (with total cost of 373 Million Rials), (b) simultaneous implementation of solutions 12, 3, and 6 (with total cost of 493 Million Rials), and (c) implementation of all solutions (with total cost of 62623 Million Rials). According to Fig. 17, when more solutions are adopted, the duration of recovery time is significantly reduced from 155 h to 120, 90, and 80 h, respectively. Restoration efficiency index (REI) defined in (15), is the proportion of the area under the restoration path to the total area under the nominal (initial) operation line within the restoration phase. By differentiating these two areas in the restoration phase, the recovery time can be obtained. In addition, Table 8 compares the resiliency assessment metrics of the base case with the cases in which multiples solutions are implemented simultaneously. According to this table, the adoption of more solutions results in enhanced resiliency metrics in exchange for higher implementation costs.

**Table 8:** Comparison of resiliency assessment metrics for Shourkand feeder in the base case with the cases of adopting multiple solutions.

Case	*VI*	*DI*	*REI*	*RI*
Bae case	1	1	0.22	0.25
Solutions 12 and 3	1	1	0.65	0.63
Solutions 12, 3, and 6	1	1	0.7	0.71
All solutions	1	1	0.74	0.74

**Fig. 18:**
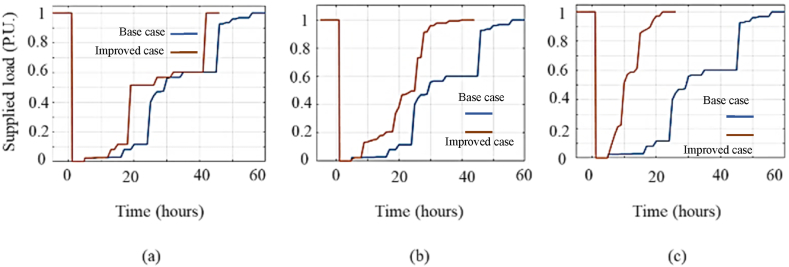
Illustrative comparison of Miandouab feeder resiliency curve for the base case and (a) implementation of solutions 12 and 6, (b) implementation of solutions 12, 6, and 1, and (c) implementation of all solutions.

Similar results can be obtained for Meandoab feeder by the simultaneous implementation of multiple solutions to improve resilience. Fig. 18 compares the resiliency curve of the Miandouab feeder in the base case plus (a) simultaneous execution of solutions 2 and 6 (with total cost of 180 Million Rials), (b) simultaneous execution of solutions 12, 6, and 1 (with total cost of 430 Million Rials), and (c) implementation of all proposed solutions (with total cost of 62623 Million Rials). Accordingly, adopting multiple solutions decreases the recovery process to 45, 35, and 25 h in Fig. 18 (a)–(c). In addition, Table 9 compares the resiliency indices of the base case with the cases of multiple solutions. It is confirmed that adoption of more enhancement measures results in a more resilient feeder in exchange for higher implementation cost.

**Table 9:** Comparison of resiliency assessment metrics for Miandouab feeder in the base case with the cases of adopting multiple solutions.

Case	VI	DI	REI	RI
Base case	1	1	0.22	0.30
Solutions 12 and 6	1	1	0.61	0.55
Solutions 12, 6, and 1	1	1	0.68	0.6
All solutions	1	1	0.74	0.66

### 5.4 Discussion

This section discusses the results of implementing different resilience enhancement solutions in the previous section. In Section 5.2, first, the proposed resiliency assessment method was implemented on Shourkand and Miandouab feeders, which are highly vulnerable to salt dust due to their proximity to the over-saturated salt Urmia Lake. The initial resiliency levels of these feeders were quantified, with Shourkand and Miandouab feeders showing degraded resiliency levels of 0.25 and 0.30, respectively, in their current conditions (Table 4, Table 5). Second, the study then prioritized resiliency-enhancement measures based on their BCR index. The following discusses those solutions and their impact on improving the resilience of the studied distribution feeder.

The results indicate that incorporating cut-out fuses into the network significantly reduces the recovery time, for example to 25 h for Miandouab Feeder. The recovery time for Miandouab feeder was around 60 h in the base case. This improvement is attributed to the ability of cut-out fuses to expedite the reconnection of any repaired lines to the network, thereby swiftly restoring power to the connected loads. Consequently, the overall recovery time is minimized. In addition to enhancing recovery speed, cut-out fuses present a high economic advantage due to their relatively low cost compared to other protective solutions. This cost-effectiveness makes them an attractive option for improving network resiliency.

Similarly, cabling the lines also results in a reduction of network recovery time to approximately 25 h. However, this method entails a significantly higher investment cost, making it less economical compared to the use of cut-out fuses. Based on the results presented in Table 5, Table 7, it is evident that in both the Meandouab and Shorkand networks, the installation of cut-out fuses is more economical, while the cabling of the lines is not cost-effective. Other solutions fall between these two approaches in terms of both recovery time and investment cost. While they may offer varying degrees of effectiveness and cost, the balance between recovery time and economic viability positions cut-out fuses as a highly advantageous method for enhancing network resiliency.

Based on the simulation results for the Meandoab and Shorkand feeders, it is evident that washing the contaminated insulators significantly reduces the network recovery time. Specifically, the recovery time for the Shorkand feeder is reduced to 72 h, while for the Meandoab feeder, it is reduced to 30 h. The reduction in recovery time is primarily due to the removal of accumulated salt and other pollutants from the insulator surfaces. By eliminating these contaminants, the risk of electrical faults caused by conductive paths is minimized, thereby enhancing the overall reliability of the power system. Clean insulators maintain their insulating properties more effectively, which prevents leakage currents and potential flashover incidents. This proactive maintenance strategy ensures that the insulators function optimally, leading to quicker restoration of power in the event of outages. Moreover, the improved condition of the insulators translates to fewer electrical discharges and faults, resulting in a more stable and reliable power supply. This stability is crucial for reducing downtime and ensuring that power is restored swiftly following any interruptions. The significant reduction in recovery time for both feeders demonstrates the effectiveness of regular washing in maintaining network performance and resilience. Additionally, the economic benefits of washing insulators are notable. By preventing faults and reducing recovery times, the need for extensive repairs and maintenance is minimized. This reduction in maintenance activities lowers operational costs and enhances the economic efficiency of the power system. The simulation results highlight that investing in regular washing of insulators is a cost-effective strategy that yields substantial benefits in terms of reliability and resilience. To avoid redundancy, the following discusses the impact of the remaining proposed solutions to enhance system resilience in the studied distribution feeders.

• Solution 1

Allocating 8 fault detectors in the distribution system has a significant impact on improving power system resilience. These detectors enable the early identification of faults within the network, allowing for quicker response and resolution. By detecting faults promptly, the system can isolate and address issues faster, reducing the duration and impact of outages. Additionally, accurate fault detection helps maintenance crews target specific problem areas, enhancing the efficiency of repair and maintenance activities. Based on the studied feeders, by adding 8 fault detectors, the recovery time is reduced to 70 and 35 h for Shourkand and Miandouab feeders, respectively. The improvement in recovery time is reflected in the improvement of the resilience indexes for these feeders too. Solution 1 increases the RI index for these feeders to 0.27 and 0.42, respectively.

• Solution 3

Replacing 400 insulators enhances the insulation performance of the system. New insulators typically have improved material properties, such as better hydrophobicity and resistance to pollution, which reduces the risk of electrical failures. This replacement decreases the likelihood of flashovers and other insulator-related faults, improving overall system reliability. Furthermore, new insulators extend the operational life of the infrastructure, delaying the need for further replacements and reducing maintenance costs. As an example, by replacing 400 insulators, the recovery time is reduced to 75 and 25 h for Shourkand and Miandouab feeders, respectively. Resilience indexes for these feeders also improved as a result of the improvement in recovery time. In Solution 3, the RI index for these feeders is increased to 0.37 and 0.31, respectively.

• Solution 4

Adding another crew team increases the capacity for responding to faults and maintenance needs, reducing the time required to address issues. More crews enable more extensive and frequent maintenance activities, improving the overall condition of the network. With more resources available, the power system can handle multiple simultaneous faults or emergencies more effectively, thereby increasing resilience. As an example, by adding another crew team, the recovery time is reduced to 110 and 29 h for Shourkand and Miandouab feeders, respectively. In addition to improving recovery time, the resilience indexes for these feeders have also improved. RI indexes for these feeders are increased to 0.27 and 0.34, respectively, in solution 4.

• Solution 5

Using emergency distributed energy resources (DERs), such as backup diesel generators, provides a reliable backup power source during outages, ensuring continuous supply to critical loads. The availability of backup generators enhances the system's ability to withstand and recover from major disruptions or faults. DERs reduce the reliance on a single power source, diversifying the energy supply and improving overall system reliability. For example, although adding a crew team in Solution #4 reduces network recovery time, it requires hiring additional personnel and incurs higher costs. Therefore, this method is less advantageous compared to the installation of cut-out fuses. In both the Miandouab and Shurkand networks, the addition of a crew team is ranked in the middle of the table in terms of cost-effectiveness. As an example, by using DERs, the recovery time is reduced to 75 and 25 h for Shourkand and Miandouab feeders, respectively. Moreover, the improvement in recovery time is reflected in the improvement of resilience indexes for these feeders as well. By implementing solution 5, the RI index for these feeders is increased to 0.45 and 0.31, respectively.

• Solution 6

Using canopies for transformers provides substantial benefits. Canopies act as protective shields that prevent direct exposure of transformers to salt dust, which can cause corrosion and degradation over time. By shielding transformers from salt contamination, canopies enhance their longevity and reduce the frequency of maintenance and replacement. While the initial investment for installing canopies may be higher, the long-term cost savings and improved durability of the equipment justify this expense. The ease of installing canopies further supports their prioritization, as they can be implemented without major disruptions to the network. As an example, by using canopies for transformers, the recovery time is reduced to 45 and 26 h for Shourkand and Miandouab feeders, respectively. Improvements in recovery time also result in improved resilience indices for these feeders. Using solution 6, the RI indexes for these feeders are increased to 0.27 and 0.35, respectively.

• Solution 8

Extending maneuver points with adjacent feeders improves fault management by allowing for more flexible reconfiguration of the network. This facilitates the isolation of faulty sections and rerouting of power, enhancing load balancing and reducing the risk of overloading. Better maneuverability enables more efficient load distribution, reducing the risk of overloading and improving system stability. More maneuver points help limit the affected areas during faults, minimizing the impact on customers and enhancing resilience. As an example, by extending maneuver points with adjacent feeders, the recovery time is reduced to 90 and 28 h for Shourkand and Miandouab feeders, respectively. Improved recovery time reflects improved resilience indexes for these feeders as well. It can be seen that solution 8 increases the RI index for these feeders upwards to 0.36 and 0.32, respectively.

• Solution 9

Installing three remote-controlled switches (RCSs) enhances operational flexibility, allowing operators to remotely control and reconfigure the network. This capability enables quick isolation of faulty sections and restoration of service, reducing downtime. Remote control capabilities also facilitate better load management and fault handling, improving the overall resilience of the distribution system. As an example, by installing three RCSs, the recovery time is reduced to 80 and 25 h for Shourkand and Miandouab feeders, respectively. As a consequence of the improvement in the recovery time, the resilience indexes for these feeders have also improved, and their resilience has improved as well. In Solution 9, the RI index for these feeders is increased to 0.3 and 0.36, respectively, increasing the RI index for these feeders.

• Solution 10

Replacing ten aerial distribution substations with pad-mounted ones provides improved protection from environmental factors such as wind, rain, and wildlife, reducing the risk of faults. These substations are also more secure against vandalism and accidental damage, improving overall reliability. The enhanced protection from the elements and reduced physical stress extend the operational life of the equipment. As an example, by replacing ten aerial distribution substations, the recovery time is reduced to 40 and 23 h for Shourkand and Miandouab feeders, respectively. Recovery time improvements are also reflected in an improvement in resilience scores for these feeders. In solution 10, we increase the RI index for these feeders to 0.52 and 0.35, respectively, for these feeders.

• Solution 11

Using aerial bundled cables significantly reduces faults caused by external interference, such as tree branches, animals, and other factors. These cables improve safety by reducing the risk of electrical hazards, such as accidental contact, and contribute to a more reliable and resilient power distribution system by minimizing the risk of faults. As an example, by using aerial bundled cables, the recovery time is reduced to 70 and 29 h for Shourkand and Miandouab feeders, respectively. It is clear that the improvement in the recovery time has also led to an improvement in the resilience indexes for these feeders as well. In Solution 11, the RI index and RI value for these feeders are increased to 0.27 and 0.42, respectively.

## 6 Conclusion

Iran is faced with different natural disasters and extreme weather events such as dust storms. Therefore, the power system resiliency should be improved in face of such HILP incidents. The study investigated the resilience of distribution networks in Iran against salt dust through a detailed analytical approach. First, the study developed and executed an experimental procedure in the High Voltage Laboratory at the University of Tehran to extract three-dimensional fragility curves against salt pollution and relative humidity for different types of insulators. The experimental results, as depicted in Fig. 3, Fig. 4, demonstrated that polymeric composite insulators are best suited for regions with a high risk of salt dust, affirming the necessity of selecting appropriate insulators based on geographical and environmental conditions. Second, the study leveraged the expert knowledge of managers and operators from the West Azerbaijan electric distribution company to propose solutions for enhancing the resiliency of the feeders under study. This collaborative approach ensured practical relevance and applicability of the suggested measures. Third, the proposed resiliency assessment and improvement method was implemented on Shourkand and Miandouab feeders, which are highly vulnerable to salt dust due to their proximity to the over-saturated salt Urmia Lake. The initial resiliency levels of these feeders were quantified, with Shourkand and Miandouab feeders showing degraded resiliency levels of 0.25 and 0.30, respectively, in their current conditions (Table 4, Table 5). The study then prioritized resiliency-enhancement measures based on their BCR index. The most efficient measure was the addition of six cut-out fuses, followed by using canopies for transformers, allocating fault detectors, and replacing/washing insulators (Table 5, Table 7). These measures were evaluated for their cost-effectiveness, confirming that significant resiliency improvements could be achieved while minimizing investment. For example, the resiliency metric RI for the Shourkand feeder increased from 0.25 in the base case to 0.63 by implementing these measures (Table 6). The probabilistic nature of the proposed method allows for a comprehensive assessment of different resilience enhancement strategies. Monte Carlo simulations could further capture the probabilistic failure mechanisms of insulators, although this would increase computational demands. However, since the presented framework focuses on resiliency-oriented planning, the added computational burden is considered a manageable aspect of the study. The findings of this study are vital for distribution system managers in conducting budget allocation studies and implementing the most efficient measures for improving system resiliency. The methodology demonstrated here can be a valuable tool for enhancing the performance of distribution systems in similar high-risk regions. Further work is necessary to establish a methodology that analyzes the mutual effects of different resiliency improvement approaches on overall resiliency. Additionally, the integration of Monte Carlo simulations could refine the analysis by incorporating more detailed probabilistic considerations, albeit with increased computational effort.

## Data availability statement

Data will be made available on request.

## CRediT authorship contribution statement

**Amin Dadashzade:** Writing – original draft, Software, Methodology, Investigation, Conceptualization. **Hossein Bagherzadeh:** Visualization, Software, Methodology, Formal analysis. **Masood Mottaghizadeh:** Software, Resources, Methodology, Formal analysis. **Tohid Ghanizadeh Bolandi:** Supervision, Project administration, Investigation, Conceptualization. **Mohammad Hassan Amirioun:** Writing – review & editing, Validation, Investigation. **Maryam Majidzadeh:** Visualization, Investigation, Data curation. **Sajjad Golshannavaz:** Writing – review & editing, Validation, Formal analysis. **Farrokh Aminifar:** Validation, Investigation, Formal analysis.

## Declaration of competing interest

The authors declare that they have no known competing financial interests or personal relationships that could have appeared to influence the work reported in this paper.
